# TTV and other anelloviruses: The astonishingly wide spread of a viral infection

**DOI:** 10.1016/j.amolm.2023.100006

**Published:** 2023

**Authors:** Pietro Giorgio Spezia, Daniele Focosi, Andreina Baj, Federica Novazzi, Francesca Drago Ferrante, Fabrizio Carletti, Claudia Minosse, Giulia Matusali, Fabrizio Maggi

**Affiliations:** a Department of Translational Research, University of Pisa, Italy; b North-Western Tuscany Blood Bank, Pisa University Hospital, Pisa, Italy; c Department of Medicine and Surgery, University of Insubria, Varese, Italy; d Laboratory of Virology and Biosafety Laboratories, National Institute of Infectious Diseases “L. Spallanzani” IRCCS, Rome, Italy

**Keywords:** TTV, Anellovirus, TTMV, TTMDV, Virome, Immune system

## Abstract

The broad family of viruses known as anelloviruses (AV) infects both humans and numerous animal species. They have a tiny, covalently closed single-stranded DNA genome and the astonishing capacity to infect a very high percentage of healthy and ill people with chronic infections that could last a lifetime. AV, and particularly the prototype Torquetenovirus, have established a successful interaction with the host's immune system and the rate at which they replicate is a gauge to measure overall immune function, even though many aspects of their life cycle and pathogenesis are still poorly understood.

## 1 Introduction

**Fig. 1:**
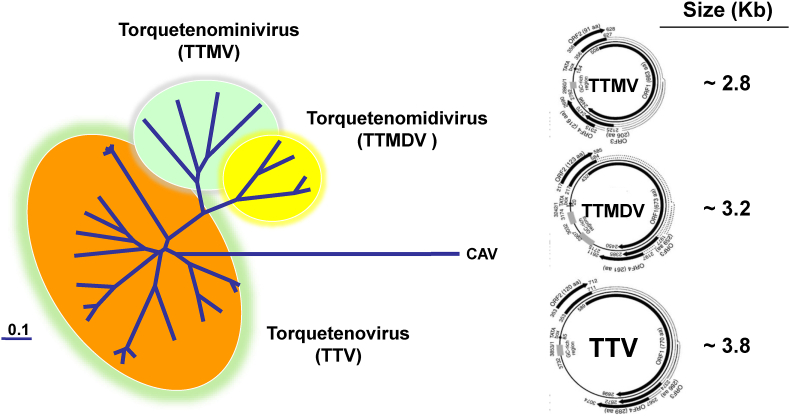
Schematic representation of the viral species of Anelloviridae.

**Fig. 2:**
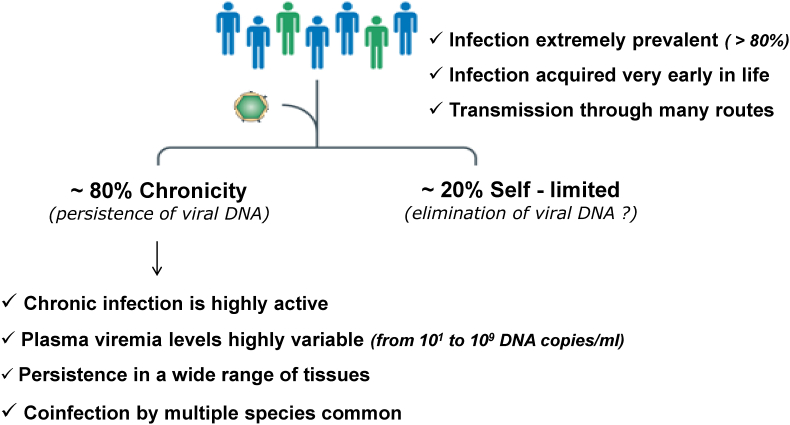
Relevant aspects in the epidemiology and pathogenesis of TTV infection.

Anelloviruses (AV) comprise a sizable genus of pathogens that infect both humans and numerous animal species. When a Japanese team used representational difference analysis to look for novel hepatotropic viruses in the blood of patients with cryptogenetic post-transfusion hepatitis, it provided the first evidence of AV existence (Nishizawa et al., 1997; Okamoto et al., 1998). The team discovered a novel virus with an unusually tiny genome made of negative-polarity, single-stranded circular DNA. This new virus was initially thought to be a novel hepatitis virus and, from the initials of the first patient in whom it was discovered, it was named TT virus (TTV). TTV's significance changed in 2004 when it became the abbreviation for torquetenovirus (from the Latin words torques and tenuis, meaning necklace and thin, respectively) for adhering to the International Committee on Taxonomy of Viruses' regulation that no official virus designation may be taken from a person's name. The discovery of TTV also unlocked a variety of interesting functions for viruses. As soon as the virus was discovered, it became evident that TTV was neither linked to hepatitis nor to any other recognized diseases because it was shown to be common in both healthy and sick individuals. TTV was followed in the year 2000 by many related, previously unidentified viruses that had genomic characteristics similar to, but occasionally extremely different from those of TTV. Torquetenominivirus (TTMV) and Torquetenomidivirus (TTMVD) were the names of those virus' subgroups (Takahashi et al., 2000; Ninomiya et al., 2007). Since 2009, all these viruses have been grouped under the family Anelloviridae (from the Latin word anellus, which means "ring" to denote the circular genome). AV have been proven to be remarkably prevalent, with abundant viral DNA identified in the plasma of 80% or more of the world's population, but their importance for human health is unknown. Recent evidence demonstrating that AV are the most prevalent members of the human virome speaks in favor of the concept that they should be entirely non-pathogenic, but probably it's wiser considering them as "orphans of disease," as has been done with other viruses that were found to cause substantial diseases after a long period of time from their discovery (see Fig. 1, Fig. 2).

## 2 Classification

AV were initially classified as a member of the Circoviridae family. Further sequencing of AV genomes revealed that, despite sharing many genetic traits with this family, they diverged significantly from circoviruses and lacked significant sequence homology. Thus, the novel Anelloviridae family was created because of the identification of these distinctions and other features. To date, the Anelloviridae family is divided into 31 genera each of which is denoted by a letter of the Greek alphabet (Table 1) (Varsani et al., 2943). Withing the genera, there are 155 species. Human AV are mainly classified into three genera: *Alphatorquevirus* (which identifies TTV sequences), *Betatorquevirus* (which identifies TTMV sequences), and *Gammatorquevirus* (which identifies TTMDV sequences). The genetic classification of human TTV sequences includes at least 22 major species, each of which includes many isolates. AVs in the genus *Gyrovirus* have been identified infecting various avian species where those on the remaining 30 genera have been found primarily various infecting mammal species.

**Table 1:** List of genera and species in the family of Anelloviridae.

Family	Genus	No. of species included	Representative species
Anelloviridae	*Aleptorquevirus*	2	Torque teno arthrovec virus
	*Alphatorquevirus*	26	Human torquetenovirus
	*Betatorquevirus*	38	Human torquetenominivirus
	*Chitorquevirus*	1	Torque teno indri virus
	*Dalettorquevirus*	1	Torque teno ursid virus
	*Deltatorquevirus*	1	Torque teno tupaia virus
	*Epsilontorquevirus*	1	Torque teno tamarin virus
	*Etatorquevirus*	5	Torque teno felis virus
	*Gammatorquevirus*	15	Human torquetenomidivirus
	*Gimeltorquevirus*	1	Giant panda anellovirus
	*Gyrovirus*	10	Chicken anemia virus
	*Hetorquevirus*	1	Torque teno hominid virus
	*Iotatorquevirus*	1	Torque teno sus virus 1
	*Kappatorquevirus*	2	Torque teno sus virus k2
	*Lambdatorquevirus*	6	Torque teno pinniped virus
	*Mutorquevirus*	1	Torque teno equid
	*Nutorquevirus*	1	Torque teno pinniped virus
	*Omegatorquevirus*	2	Gorilla anellovirus
	*Omicrontorquevirus*	1	Giant panda anellovirus
	*Pitorquevirus*	6	Torque teno ursid virus
	*Psitorquevirus*	1	Torque teno procyo virus
	*Rhotorquevirus*	1	Torque teno rodent virus
	*Sigmatorquevirus*	2	Torque teno gazella virus
	*Tautorquevirus*	1	Torque teno felid virus
	*Tettorquevirus*	1	Torque teno felid virus
	*Thetatorquevirus*	10	Torque teno canis virus
	*Upsilontorquevirus*	7	Torque teno procyo virus
	*Wawtorquevirus*	6	Torque teno rodent virus
	*Xitorquevirus*	2	Torque teno chiroptera virus
	*Zayintorquevirus*	2	Torque teno viverrid virus
	*Zetatorquevirus*	1	Torque teno douroucouli virus

## 3 Virion structure and genome

The TTV particle has a diameter ranging from 30 to 32 ​nm (below 30 ​nm for TTMV) and is roughly spherical (Maggi and Bendinelli, 2009, 2010; Bendinelli et al., 2001; Arze et al., 2021). The virus has been determined not to have an exterior lipid envelope and to be as stable to inactivation by chemical and physical agents. The genome is the part of AV that is currently best understood. Early analysis revealed that the viral genome is single-stranded DNA and it was thought to be linear when the prototype Japanese isolate had its genome sequenced to a level of roughly 90%. Then, the two extremities of the genome were found to be connected by a guanine and cytosine (GC)-rich stretch of about 100 nucleotides (nt), which was difficult to amplify and sequence in earlier studies and forms a covalently closed circular molecule. Virus encapsidation of the minus strand was also demonstrated. Though all AV genomes are similar structurally, TTV genomes range in size from 3.5 to 3.9 kilobases (Kb), TTMDV to 3.2 ​Kb, and TTMV from 2.7 to 2.9 ​Kb. All of them have a 1.2 ​kb UTR and a 2.6 ​kb coding area. The UTR possesses a short nt segment that is highly conserved among all AV isolates, and it appears to control virus replication and expression through several significant regulatory sequences and secondary structures. Two significant potential protein-coding genes, ORF1 and ORF2, make up the coding area. These ORFs are in various reading frames, have variable sizes, and are located on the plus strand, which is complementary to the genomic DNA. It is thought that the putative capsid protein is encoded by ORF1. A potential non-structural protein between 100 and 120 amino acids and with phosphatase activity is hypothesized to be encoded by the smaller ORF2, which is important in viral replication. Sequence analysis of AV isolates forecasts the presence of additional potential ORFs that may encode poorly known proteins.

## 4 Life cycle

Even though TTV was discovered more than twenty-five years ago, the AV life cycle is not well understood, and our current understanding of replication mechanisms is largely based on extrapolation from what is known about other single-stranded DNA viruses. TTV is known to infect various cell types and can be found throughout the body. Therefore, it is assumed that the cellular receptor(s) required for viral entry are molecule(s) present in large quantities in body tissues and are exposed by a diverse range of cell types. There is currently no precise characterization of the cell types supporting viral replication. It is plausible that most, if not all, AV viruses depend on cellular proteins generated during the S-phase of the cell cycle for productive reproduction, as similar single-stranded DNA viruses do (Maggi and Bendinelli, 2009; Bendinelli et al., 2001; J Taylor et al., 2022; Kaczorowska and van der Hoek, 2020). Additionally, it has been demonstrated that a single cell can harbour many AVs, which helps to explain why recombination is so common among these viruses. Three different-length RNA transcripts have been found of 3.0, 1.2, and 1.0 ​kb in size, respectively. Although the exact mechanism of DNA replication is unknown, there are various signs (i.e., ORF1 contains conserved Rep protein motifs) that point to a rolling, circular mechanism, and that the genome is replicated by cellular enzymes. MicroRNA-coding regions have been found in a variety of human TTVs, probably creating TTV microRNA in vivo (Vignolini et al., 2016). Unknowns are virion assembly and release mechanisms from producer cells. According to what has been observed with other nonenveloped viruses, virus particles are likely formed in the cytoplasm and released by cell lysis. However, recent research showing that TTV particles are contained within circulating extracellular vesicles provides alternate pathways for progeny virions to exit the body. The ability of TTV particles to circulate into extracellular vesicles could represent a mechanism for avoiding the immune host response making the virus less exposed to neutralizing antibodies (Martelli et al., 2018).

## 5 Epidemiology

The astonishingly widespread infection in the general community and around the world is a well-documented aspect of AV epidemiology. Nearly two-thirds of the general population are infected with TTV (Spezia et al., 2022; Shulman and Davidson, 2017; Väisänen et al., 2022; Spandole et al., 2015). However, AV could be even more prevalent, reaching 100%, when subjects without viremia but harbouring the viruses in other body tissues are considered. It's interesting to note that the prevalence is equally high in groups of people who live in isolation and have little contact with the outside world. People who have multiple AV in their blood and/or other tissues are more often the rule than the exception because TTMV and TTMDV have a prevalence of current infections equivalent to TTV. Even though some studies show that viremia rates rise with age and reach their peak at maturity, AV infection is common even in the first few months of life. This suggests that an AV infection is extremely infectious and spreads through a variety of channels. It has been shown that blood and blood components can transmit AV. TTV is frequently found in many other biological fluids (saliva, faeces, urine, genital secretions, tears, nasal fluid), thus suggesting that multiple methods of transmission exist. TTV is also vertically transmitted with a mother-to-child transmission rate ranging from 67% to 14% (Morrica et al., 2000; Xin et al., 2004). It is unknown if non-human AV or AV-like viruses, which have been found to be common in pigs, chickens, cats, dogs, and other farm and companion animals, contribute to the epidemiology of AV in humans. Interestingly, high prevalence rates of TTV were found in common murine rodents, thus suggesting that these animals could act as a natural reservoir for the virus, and they could represent ideal models to investigate the transmission, virulence, immunity, and pathogenesis of AV (Xiong et al., 2018).

Of utmost importance, among the aspects that are still poorly understood, are the immunological properties that make the AVs so successful at evading control by the host's innate and adaptive defences. Based on what is known, it seems safe to assume that the adaptive immune responses mounted by the infected hosts play a key role both in determining whether a primary AV infection will resolve and in setting the extent to which the AVs circulate in the peripheral blood of those who fail to clear them. To this purpose, the contribution of some cells, such as B-cells, T-cells, natural killer cells and antigen-presenting cells, in virus control has been demonstrated. Furthermore, recent studies have suggested that AV could evade the host antibody response by excision of the immunodominant ORF1 C terminus from the mature virus and that many AV peptides do not elicit a detectable antibody reactivity, thus favouring the immune evasion of these commensal viruses (Venkataraman et al., 2022; Liou et al., 2022; Moen et al., 2003).

Information on the consequences of AVs replication on infected cells is also poorly understood. Evidence exists that the ORF2 protein of TTV has the potential to suppress the activity of NF-κB, a well-characterised intracellular signal transcription factor known to promote the expression of many genes including pro-inflammatory cytokines, chemokines, adhesion molecules, inducible enzymes, and immune receptors. Thus, TTV, and probably other AV species, are potentially capable of having a significant impact, by means of the ORF2 protein, many factors that concur at effecting or regulating the inflammatory response. Moreover, the TTV DNA was found to provoke a robust activation of TLR-9 in ex vivo-grown mouse spleen cells, as revealed by the production of pro-inflammatory cytokines, thus suggesting TLR-9 as one of the cell sensors through which replicating AVs may impact the inflammatory response in the host (Rocchi et al., 2009; Zheng et al., 2007; Kakkola et al., 2002, 2008; Chen et al., 2013).

## 6 Clinical features

TTV and AV in general are currently unknown in terms of their medical importance. Undoubtedly, the circumstances surrounding TTV's discovery had a significant impact on early research that sought to connect the virus to a clinical condition. The apparent ability of TTV to replicate in the liver and the temporal correlation between TTV viremia and transaminase elevations seen in transfused patients in whom TTV was demonstrated prompted the virus to be implicated as a potential cause of acute and chronic liver diseases. Later research refuted this association, and although it cannot be ruled out that TTV infection could occasionally be linked to liver damage of varying degrees, the virus is not currently believed to be the cause of this liver damage. As a result, efforts have been made to assess TTV and other AV's potential contribution to the development of extrahepatic disorders with unknown causes (Spandole et al., 2015; Webb et al., 2020; Dodi et al., 2021). Most studies have been focused on chronic conditions (diabetes, cryoglobulinemia, psoriasis, rheumatoid arthritis, systemic lupus erythematosus, Kawasaki syndrome, multiple sclerosis, respiratory diseases), however no direct contribution of TTV to illness etiology has been clearly ruled out. Thus, to date, TTV and other AV are regarded as "viruses waiting for a disease" or "orphans of illness". In fact, it cannot be excluded that only a few AV types can be pathogenic, and that the abundance of non-pathogenic kinds obscures their virulence. Whether AV affects the host's adaptive immune response is still an open question. It has also been demonstrated that the TTV genome and its intermediate form of replication in infected cells may affect the production of pro-inflammatory cytokines induced by Toll-like receptor stimulation. More recent studies have revealed that TTV is a crucial component of the human virome. Virome is a term used to describe the collection of viral species present in a human organ. Viruses, once thought to be present only in the host during disease, have recently been shown to be abundant in many body regions of healthy people. This kind of viral "flora" consists of a collection of "harmless" organisms like bacteriophages, endogenous retroviruses, harmless eukaryotic viruses, and harmful viruses that can cause latent, chronic, or acute disorders. Currently, it is known that some components of the human virome are distributed throughout practically all body sites in a very high percentage of people, whereas others are only found in a small number of communities and individuals. The most prominent instance of the latter is AV, and more specifically, TTV. They are the most prevalent and representative viruses in the human virome, and their load in plasma is suggested as a straightforward and all-encompassing indicator of how well-infected hosts' immune systems are functioning. According to research, the measure of TTV viremia may be helpful in determining the immunological state of those who have received solid organ transplants and in monitoring immunosuppression and risks of post-transplant complications (Redondo et al., 2022; Fernández-Ruiz, 2020; Jaksch et al., 2022; Maggi et al., 2018; Giacconi et al., 2018). More recently, the predictive role of TTV load with regard to vaccine response anti-SARS-CoV-2 as well as a potential pharmacodynamic biomarker in patients with chronic arthritis treated with biologic DMARD has also been demonstrated, suggesting other intriguing applications for this widespread AV (Martin-López et al., 2020; Studenic et al., 2022; Focosi et al., 2023).

## 7 Pathogenesis

The potential of AV to generate chronic productive infections in the blood of most exposed individuals, if not all, is a well-known property. Unknowns are the body locations and cell types where AV conducts initial amplification after entering the body, as well as the tissues supporting its ongoing replication and shedding in blood circulation (Focosi et al., 2020). The respiratory tract may be a source of initial TTV amplification, the virus is then detectable in peripheral blood within one or a few weeks of admission, and the most common result of TTV infection is a protracted, perhaps lifelong TTV viremia. TTV viremia is largely stable in many people over time, while it fluctuates significantly in others over time due to a variety of factors. Less is known about the remaining features of TTV's interactions with infected hosts. Most of the evidence points to hematopoietic cells that are actively growing as a significant source of circulating TTV. The fact that baseline TTV viremia dropped to undetectable levels in patients receiving myelosuppressive therapies in preparation for bone marrow transplantation supports the theory (Maggi et al., 2010). While little is known about anti-TTV cell-mediated immune responses, the evidence that is now available about humoral responses implies that TTV evokes antiviral antibodies that, at least in most instances, fail to eliminate the virus. The role of interactions with other coinfecting viruses (i.e cytomegalovirus, Epstein-Barr virus) in AV pathogenesis has been still poorly investigated.

## 8 Diagnosis

AV infection laboratory diagnosis is limited by several factors: 1) there are no trustworthy and sensitive tissue culture systems for AV isolation and propagation; 2) there are no simple immunological methods to detect and titrate AV-induced antibodies; and 3) it is also impossible to detect viral antigens in plasma. To diagnose infections, the TTV genome must be found in blood or other specimens. According to this purpose, the UTR, particularly a segment of 100 nucleotides, is highly conserved among all human TTV species and is suitable for the development of PCR assays capable of detecting all the currently known species. NGS technology is a respectable substitute for accurate genotyping. Although standardized protocols for measuring TTV viremia are still poorly developed and each laboratory independently produces its own test, recently, a standardized CE-certified PCR assay has been commercialized, and a TTV-specific independent external quality assessment (EQA) pilot program has been run by Quality Control for Molecular Diagnostics (QCMD). These tools facilitate the setup of clinical trials and the standardization of measures of TTV viremia for the identification of the TTV load cutoff to get predictions.

## 9 Prevention and treatment

There will almost certainly be little focus on how to prevent and control AV infection until their clinical implications are defined. However, according to their virological features, there is little doubt that trying to prevent infections would be extremely difficult in the absence of specific multivalent vaccines. There are no virostatic drugs with proven efficacy for AV infection, and what we know about the lack of AV pathogenicity would not justify specific clinical trials. In patients given IFN alpha alone or in association with ribavirin for HBV or HCV infections, these treatments have been seen not to abate or to only transiently abate concomitant TTV viremia (Maggi et al., 2001). Again, antiretroviral drugs have also shown no anti-TTV activity. Thus, should they one day be regarded as clinically useful, treating established infections would most likely require the development of specific antivirals.

## Funding

This work was funded by the European Union's Horizon 2020 research and innovation programme under grant agreement number 896932 (TTVguideTX project).

## Declaration of competing interest

The authors declare that they have no known competing financial interests or personal relationships that could have appeared to influence the work reported in this paper.
